# A bibliometric analysis of orthogeriatric care: top 50 articles

**DOI:** 10.1007/s00068-021-01715-y

**Published:** 2021-06-11

**Authors:** Johannes Dominik Bastian, Malin Kristin Meier, Raphael Simon Ernst, Jochen Gieger, Andreas Ernst Stuck

**Affiliations:** 1 Department of Orthopaedic Surgery and Traumatology, Inselspital, Bern University Hospital, University of Bern, Murtenstrasse, 3010 Bern, Switzerland; 2 Department of Geriatrics, Inselspital, Bern University Hospital, University of Bern, Bern, Switzerland

**Keywords:** Orthogeriatric, Co-management, Elderly, Bibliometric

## Abstract

**Background:**

Population is ageing and orthogeriatric care is an emerging research topic.

**Purpose:**

This bibliometric review aims to provide an overview, to investigate the status and trends in research in the field of orthogeriatric care of the most influential literature.

**Methods:**

From the Core Collection databases in the Thomson Reuters Web of Knowledge, the most influential original articles with reference to orthogeriatric care were identified in December 2020 using a multistep approach. A total of 50 articles were included and analysed in this bibliometric review.

**Results:**

The 50 most cited articles were published between 1983 and 2017. The number of total citations per article ranged from 34 to 704 citations (mean citations per article: *n* = 93). Articles were published in 34 different journals between 1983 and 2017. In the majority of publications, geriatricians (62%) accounted for the first authorship, followed by others (20%) and (orthopaedic) surgeons (18%). Articles mostly originated from Europe (76%), followed by Asia–pacific (16%) and Northern America (8%). Key countries (UK, Sweden, and Spain) and key topic (hip fracture) are key drivers in the orthogeriatric research. The majority of articles reported about therapeutic studies (62%).

**Conclusion:**

This bibliometric review acknowledges recent research. Orthogeriatric care is an emerging research topic in which surgeons have a potential to contribute and other topics such as intraoperative procedures, fractures other than hip fractures or elective surgery are related topics with the potential for widening the field to research.

## Introduction

The population is ageing worldwide. The Department of Economic and Social Affairs, Population Division of the United Nations reported an estimated increase of the older population (minimum 65 years of age) by a factor 2.8 from 1980 to 2020 in the World Population Prospects 2019 [1]. Accordingly, the World Health Assembly and the United Nations General Assembly finally declared in December 2020 the period 2020–2030 as the “Decade of Healthy Aging”. That global strategy and action plan “aims on using evidence-based approaches to maximise the abilities of older persons” [2]. One of the four key actions is to “deliver integrated care and primary health services that are responsive to the needs of older people”.

Over the last decades, the number of orthopaedic conditions in older people increased substantially [3–5]. For example, in Finland the incidence of pelvic fractures in older adults significantly increased from 1970 to 2013 as did the total number of hospitalizations following pelvic fractures; in addition, a 2.4-fold increase for the occurrence of pelvic fractures is predicted for the year 2030 [4]. Geriatric orthopaedic patients represent a particularly vulnerable patient group with specific demands and characteristics. Patient-centred care of this patient population requires specific medical expertise to prevent perioperative complications and to avoid loss of independence and the need for institutional care. Therefore, the optimal acute care might require shared responsibility between orthopaedic surgeons and geriatricians.

Already in 1978, an “Orthogeriatric Unit” opened at the Queens Medical Centre in Nottingham to meet the special needs of older patients with proximal femur fractures [6]. Recently, Mukherjee et al. published a systematic review and concluded that, compared to usual care, orthogeriatric care was superior with regard to functional outcomes and the prevention of hospital acquired ulcers. The authors concluded that there is a need for more high-quality research, and conditionally recommended an orthogeriatric care model to improve outcomes in geriatric patients with isolated hip fractures [7].

Over the last years, orthogeriatric care has emerged as a new key topic in clinical research with a high number of publications reporting results of studies exploring care of the geriatric orthopaedic patient. However, a bibliometric analysis in the field of orthogeriatric care has not been published in the literature to our knowledge until to date. Thus, the aim of the presented study was to report in a bibliometric review about the current research literature on orthogeriatric care. We were interested to know, what disciplines are active in research in this field, in what geographical regions this research has been conducted, and what key topics are addressed in the most highly cited research articles about orthogeriatric care.

## Methods

From the Core Collection databasis in the Thomson Reuters Web of Knowledge, we searched for the most influential original articles with reference to orthogeriatric care. Orthogeriatric care was defined as the management of patients with an orthopaedic intervention (including elective and trauma surgery) in a collaboration between orthopaedics and geriatrics. The search was conducted on 12th of December 2020 and included all available documents. The most cited 50 articles were identified by a multistep approach and then analysed for their qualities and characteristics using this bibliometric analysis. For further interpretation, parts of the obtained data were presented in relation to estimated data for the world population provided by the United Nations (United Nations, Department of Economic and Social Affairs, Population Division. World Population Prospects 2019, Online Edition. Rev. 1.) [1] and in relation to the Gross Domestic Product (GDP) per capita (in USD) as provided by the National Accounts Sections of the United Nations Statistics Division (Basic Data Selection—amaWebClient. Accessed April 6, 2021. https://unstats.un.org/unsd/snaama/Basic).

### Selection process and eligibility criteria

**Fig. 1:**
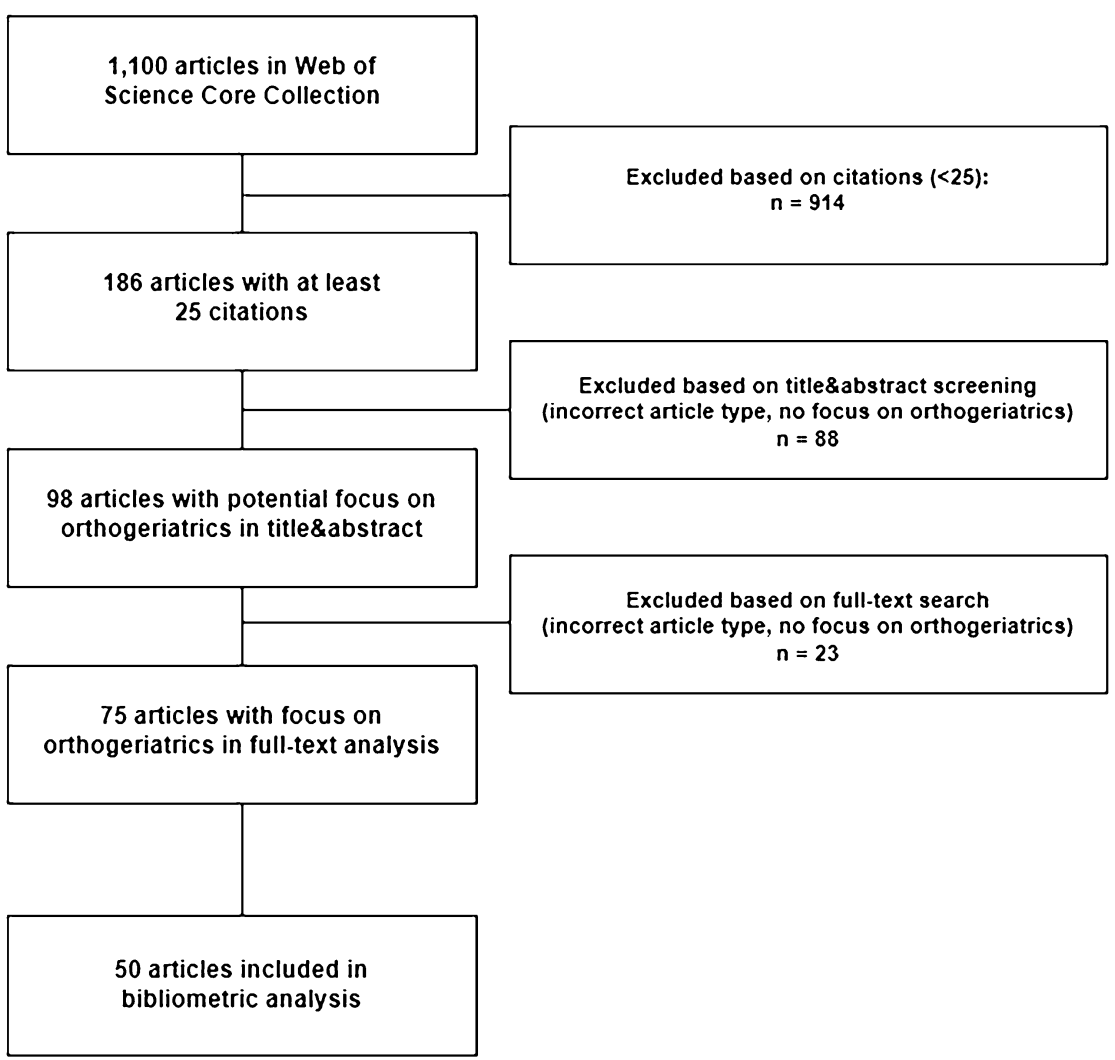
Flowchart illustrating the process of article allocation

The inclusion and exclusion of articles, as well as data extraction were conducted by a senior orthopaedic surgeon (J. D. B.) and a senior geriatrician (A. E. S.) according to predefined criteria. Disagreements between investigators were solved by consensus. The selection process was started using a title, abstract and author keywords search of the Thomson Reuters Web of Knowledge without restriction by languages and document types. The following search terms were used “orthogeriatr*” or “orthop* AND geriatr*”. The asterisk was used to extend the search, for example “orthop*” will search for orthopaedic and orthopedic. The operator “AND” was used to narrow the search. The process of inclusion and exclusion of articles is illustrated in Fig. 1. The first exclusion step was based on numbers of citations. It was assumed that relevant publications would have at least 25 citations; this was performed to reduce number of articles necessitating subsequent screening. The second exclusion step was performed in titles and abstracts based on either type of article (namely documents others than original articles, e.g. reviews, editorials, letter to the editor, case report, technical notes) or articles without focus on orthogeriatric care in patients (e.g. experimental studies, such as biomechanical studies or studies with animals, or studies describing the management of surgical patients in collaboration with geriatrics in which orthopaedic patients were only a minority of patients). This exclusion step was redone in a third exclusion step using full-text article search in remaining cases. For the final inclusion of identified articles, we ranked all articles according to their total citation rate; number one having the highest number of total citations. In case of an equal number of total citations, the articles were further ranked according to the average citation per year and then according to citations in 2020. For the bibliometric analysis, the 50 most cited articles were included.

### Data extraction and assessment

For data analysis, information available at the Thomson Reuters Web of Knowledge on Dec 12th, 2020 was used. For each included article, we extracted the following parameters: total number of citations, average number of citations per year since year of publication, affiliation of first and last author (orthopaedics, geriatrics, other), geographic origin of study population, and keywords. Articles were classified as being either (1) therapeutic, (2) prognostic, (3) diagnostic studies or (4) economic and decision analyses using the Journal of Bone and Joint Surgery American classification scheme [8]. The level of evidence was established according to the Journal of Bone and Joint Surgery American criteria with level I being the strongest and level V being the weakest level of evidence [8].

### Statistical analysis

For statistical analysis, descriptive methods were used. All obtained data are defined as number, percentage, bar chart, box-plot or line diagram. For analyses and plotting of diagrams Microsoft Excel, 2016 and the online tool Wordart Version 4.7.0 (https://wordart.com) were used. For comparison of the GDP per capita within European countries with versus without identified articles, a Mann–Whitney test was performed using GraphPad Prism (Version 9.0, GraphPad Software, San Diego, CA); level of significance *p* < 0.05.

## Results

**Fig. 2:**
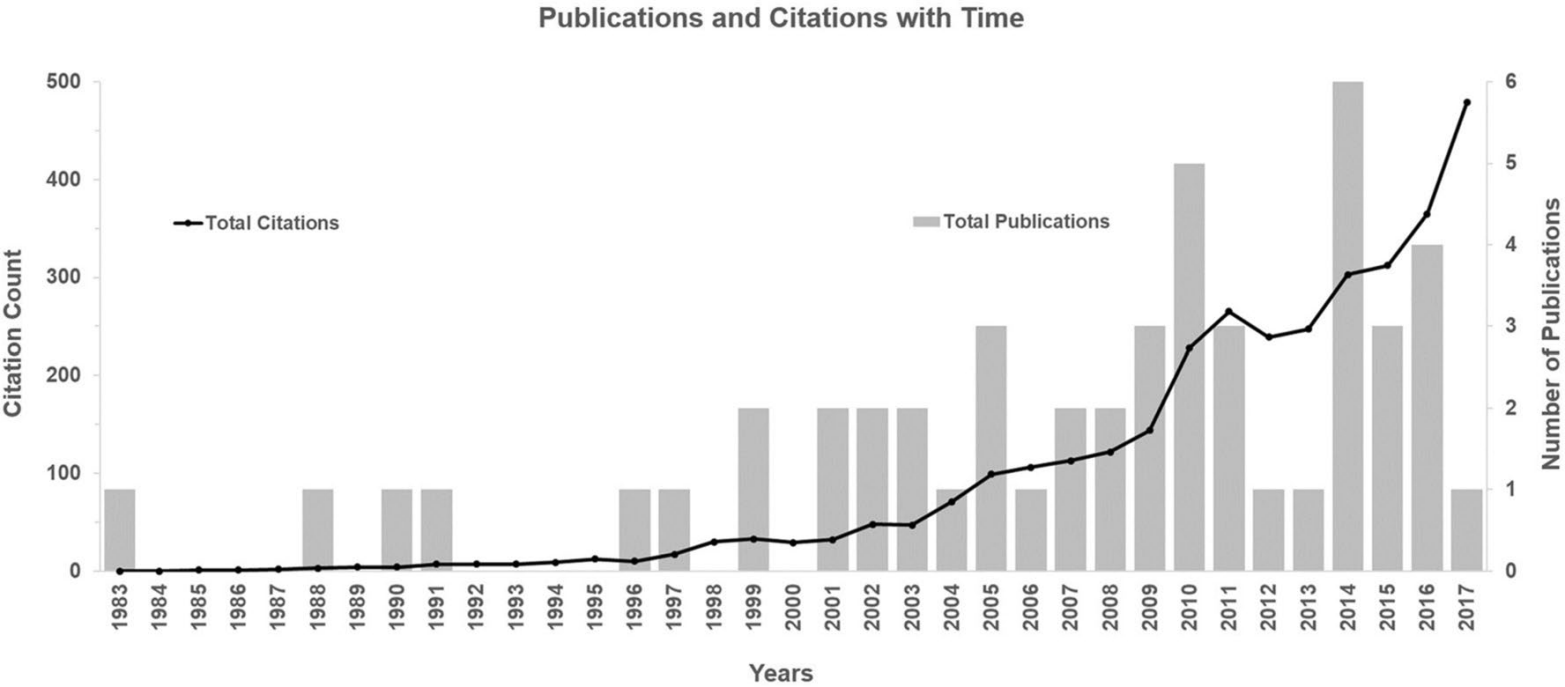
Published top 50 cited articles in each year (1983–2017) opposed to the total citation count per year

The 50 most cited articles [6, 9–57] were published between 1983 and 2017, all in English language (Fig. 2). The year with the highest number was 2014 (*n* = 6). In several years, 2000 and before (1984–1987, 1989, 1992–1995, 1998), no article among the most 50 cited ones was published. The number of total citations per article ranged from 34 to 704 citations, with a mean of 93 citations per article. The oldest study was reported in 1983 by Boyd et al. [6], the newest study was published by Folbert et al. in 2017 [22]. The average citation per year and article ranged from 1 to 39 citations, with an average of 8 citations. The citations in 2020 ranged from 0 to 53 citations, with an average of 9 citations. The highest number of total citations for all selected articles was 479 citations in 2017. The 10 most cited articles are listed in Table 1 according to the amount of total citations in descending order with authorship, title, journal and year of publication and average citations per year.

**Table 1:** List of the identified ten most cited articles in the field of orthogeriatrics listed according to the amount of total citations with average citations per year

N	Title	Total (*n*)	Average/y (*n*)
1	Marcantonio ER, Flacker JM, Wright RJ, Resnick NM. Reducing delirium after hip fracture: a randomized trial. Journal of the American Geriatrics Society 2001; 49(5): 516–22	704	35.2
2	Vidan M, Serra JA, Moreno C, Riquelme G, Ortiz J. Efficacy of a comprehensive geriatric intervention in older patients hospitalized for hip fracture: a randomized, controlled trial. Journal of the American Geriatrics Society 2005; 53(9): 1476–82	296	18.5
3	Prestmo A, Hagen G, Sletvold O, et al. Comprehensive geriatric care for patients with hip fractures: a prospective, randomised, controlled trial. Lancet 2015; 385(9978): 1623–33	236	39.3
4	Friedman SM, Mendelson DA, Bingham KW, Kates SL. Impact of a comanaged Geriatric Fracture Center on short-term hip fracture outcomes. ArchInternMed 2009; 169(18): 1712–7	226	18.8
5	Friedman SM, Mendelson DA, Kates SL, McCann RM. Geriatric co-management of proximal femur fractures: total quality management and protocol-driven care result in better outcomes for a frail patient population. JAmGeriatrSoc 2008; 56(7): 1349–56	178	13.7
6	Gustafson Y, Brannstrom B, Berggren D, et al. A geriatric-anesthesiologic program to reduce acute confusional states in elderly patients treated for femoral neck fractures. Journal of the American Geriatrics Society 1991; 39(7): 655–62	175	5.8
7	Fisher AA, Davis MW, Rubenach SE, Sivakumaran S, Smith PN, Budge MM. Outcomes for older patients with hip fractures: the impact of orthopedic and geriatric medicine cocare. Journal of orthopaedic trauma 2006; 20(3): 172–8; discussion 9–80	174	11.6
8	Lundstrom M, Olofsson B, Stenvall M, et al. Postoperative delirium in old patients with femoral neck fracture: a randomized intervention study. Aging clinical and experimental research 2007; 19(3): 178–86	168	12.0
9	Harwood RH, Sahota O, Gaynor K, Masud T, Hosking DJ, Nottingham Neck of Femur S. A randomised, controlled comparison of different calcium and vitamin D supplementation regimens in elderly women after hip fracture: The Nottingham Neck of Femur (NONOF) Study. Age Ageing 2004; 33(1): 45–51	150	8.8
10	Stenvall M, Olofsson B, Lundstrom M, et al. A multidisciplinary, multifactorial intervention program reduces postoperative falls and injuries after femoral neck fracture. Osteoporosis international: a journal established as result of cooperation between the European Foundation for Osteoporosis and the National Osteoporosis Foundation of the USA 2007; 18(2): 167–75	149	10.6

Scientists who were first author of more than 1 among the 50 most cited articles are listed in Table 2. Overall, 4 authors published 2 of the 50 most cited articles, and 1 author published 3 articles. None of these authors was a surgeon. The distribution of first authors’ specialties in relation to their contribution is depicted in Table 3. In the majority of publications, geriatricians (62%) accounted for the first authorship, followed by others (20%) and (orthopaedic) surgeons (18%). Geriatricians, being first authors, published together with senior authors being mostly geriatricians (61%) followed by others (26%; e.g. endocrinologists, epidemiologists, neuroscientists) and then by (orthopaedic) surgeons (13%). Others published with senior authors being others (60%), geriatricians (30%) or (orthopaedic) surgeons (10%). In case those (orthopaedic) surgeons were the first authors, they mainly published with senior authors being also orthopaedic surgeons (67%), followed by geriatricians (22%) and others (11%). Articles were published in 34 different journals. Journals with more than one article are listed in Table 4. Age and Ageing (The Journal of the British Geriatrics Society) published most articles (14%), followed by the Journal of the American Geriatrics Society (10%) and the Journal of the American Medical Directors Association (6%). Further four journals were identified, each with two articles (Table 4).

**Table 2:** List of authors with more than one article within the identified articles

Author	Affiliation	Years	Focus on	Articles (*n*)
Abraham Adunsky, MD	Department of Geriatric Medicine and the Orthogeriatric Unit, Sheba Medical Center, Tel-Hashomer, Israel	2003, 2005, 2011	Elderly; hip fractures; orthogeriatrics; rehabilitation; mortality	3
José I. Botella-Carretero, MD, PhD	Unit of Clinical Nutrition and Dietetics, Department of Endocrinology and Nutrition, Hospital Universitario Ramo´n y Cajal, Madrid, Spain	2008, 2010	Oral nutritional supplements; Geriatrics; Hip fracture; Surgery; Nutritional state	2
Susan M. Friedman, MD, MPH, AGSF	Department of Medicine (Division of Geriatrics), School of Medicine and Dentistry, University of Rochester, New York, USA	2008, 2009	Co-management; hip fracture; geriatrician; comorbidity	2
Juan I González-Montalvo, Prof, PhD	Geriatrics Department, La Paz University Hospital, Madrid, Spain	2010, 2016	Hip fracture, older people, orthogeriatrics, sarcopenia, Geriatric assessment, Hospital care	2
Maria Lundström, RN, PhD	Geriatric Medicine, Department of Community Medicine and Rehabilitation, Umeå University, Umeå, Sweden	1999, 2007	Nursing intervention; delirium; rehabilitation; hip fracture; delirium; femoral neck fracture; geriatric team; intervention; RCT	2

**Table 3:** Distribution of authorships in relation to specialty (in %)

	1st author		
	Geriatricians (62%)	Orthopaedics (18%)	Others (20%)
Senior author			
Geriatricians	61	22	30
Orthopaedics	13	67	10
Others	26	11	60

**Table 4:** List of journals with more than one article within the identified articles

Journal	Articles (*n*)
Age and ageing, Journal of the British Geriatrics Society	7
Journal of the American Geriatrics Society (JAGS)	5
Journal of the American Medical Directors Association, Journal of the Society for Post-Acute and Long-Term Care Medicine	3
Dementia and geriatric cognitive disorders	2
Injury, international journal of the care of the injured	2
The journals of gerontology, series A: biological sciences and medical sciences	2
Osteoporosis international	2

**Fig. 3:**
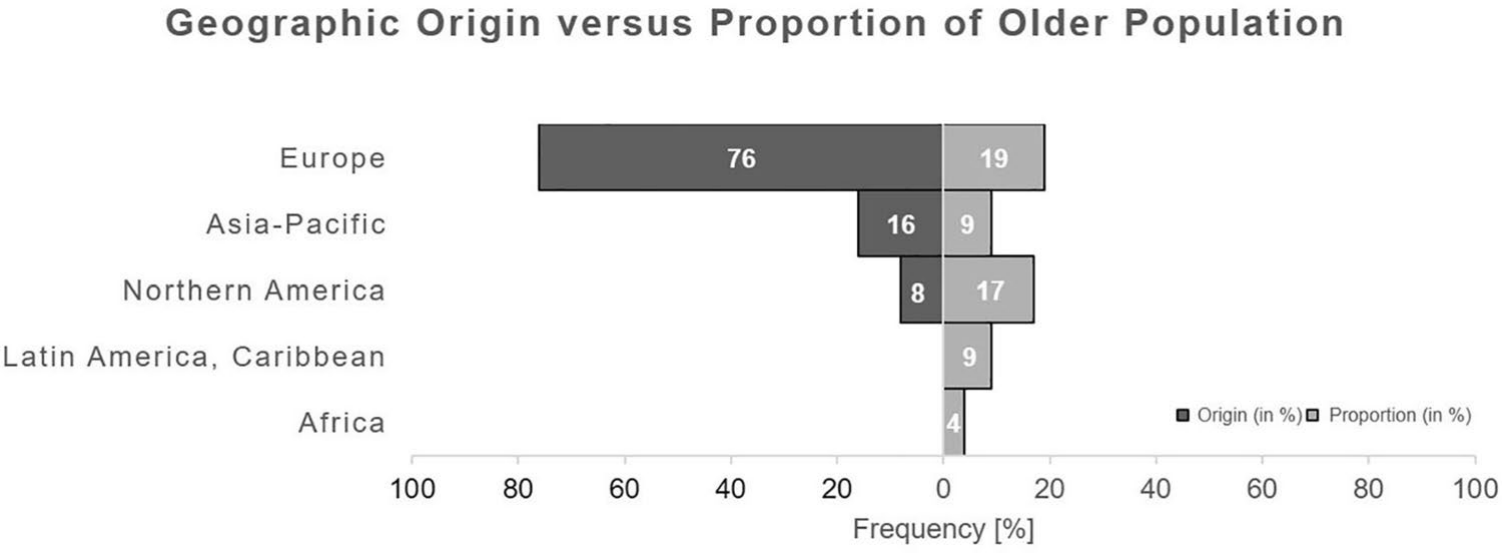
Two-sided bar chart opposing the distribution of the geographic origin of identified articles (left chart) to the proportion older (≥ 65 years) persons in the total population (right chart) within the geographic region

**Fig. 4:**
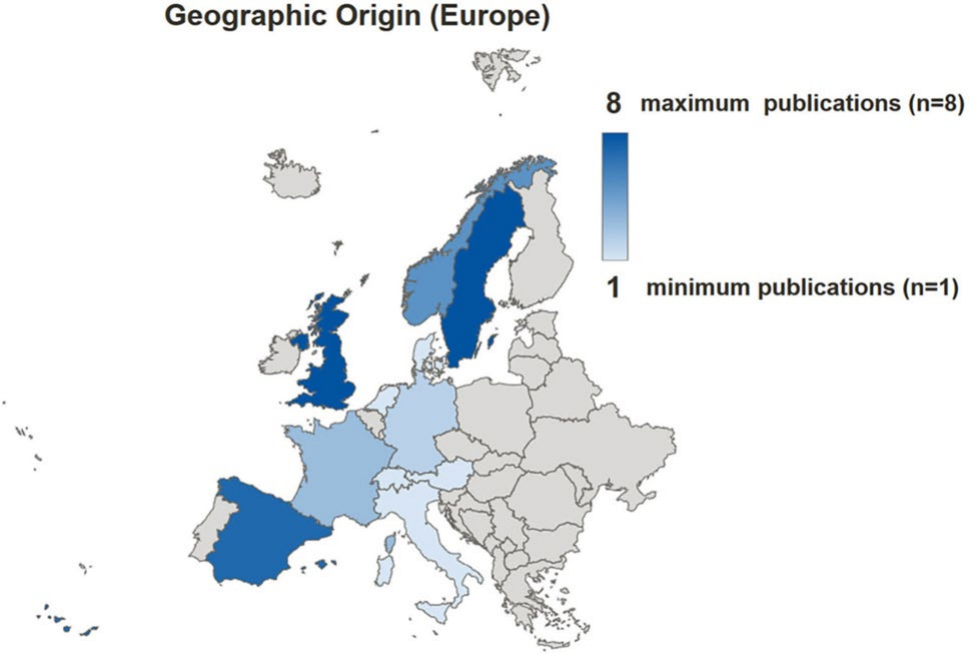
Diagram showing the geographic origin of identified articles within Europe, highlighted with light blue (least publications, minimum *n* = 1) to dark blue (most publications, maximum *n* = 8)

**Fig. 5:**
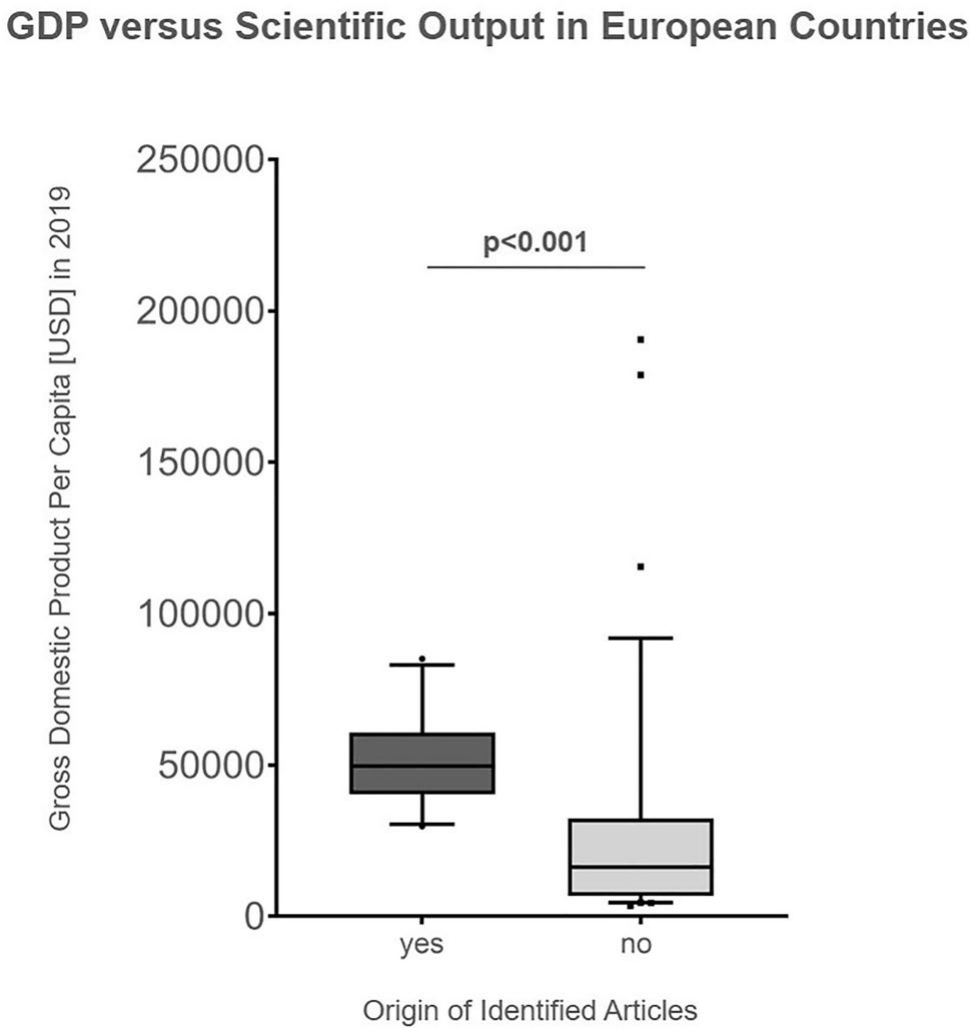
Box-plot diagram of the gross domestic product (GDP) of European countries (in USD) of countries with identified articles (*n* = 11) opposed to countries without such articles (*n* = 36). Boxes represent the inter-quartile range (IQR) and extend from the 25th to 75th percentile; whiskers are drawn down to the 10th and drawn up to the 90th percentile. Values outside the range are displayed as individual points. The line in the middle of the box represents the median. A Mann–Whitney test was performed and a significant difference (*p* < 0.001) was obtained

Identified articles mostly originated from Europe (76%) [6, 13, 15–17, 19, 20, 22, 25–36, 38, 39, 41–49, 51–57], followed by Asia–pacific (16%) [9–12, 14, 18, 21, 37] and Northern America (8%) [23, 24, 40, 50]. The analysis of the distribution of publications in relation to their geographic origin is opposed to the estimated amount of the older (at least 65 years of age) population within those different continents in Fig. 3. The highest proportion of the older population is noted in Europe (19%), followed by Northern America (17%), Asia–pacific (9%), Latin America and Caribbean (9%), and Africa (4%) (Fig. 3). Within Europe, most publications were from the United Kingdom (*n* = 8) and Sweden (*n* = 8), followed by Spain (*n* = 7), Norway (*n* = 5), France (*n* = 3) and Germany (*n* = 2); Austria, Denmark, Italy, Netherlands and Switzerland reported one article each (Fig. 4). In Europe, the GDP was statistically significant higher (*p* < 0.001; Fig. 5) in countries with identified articles (*n* = 11; median GDP: 49.701 USD; range: 29.816–85.135) compared to remaining countries without any articles (*n* = 36; median GDP: 16.303 USD; range 3.496–190.532).

**Fig. 6:**
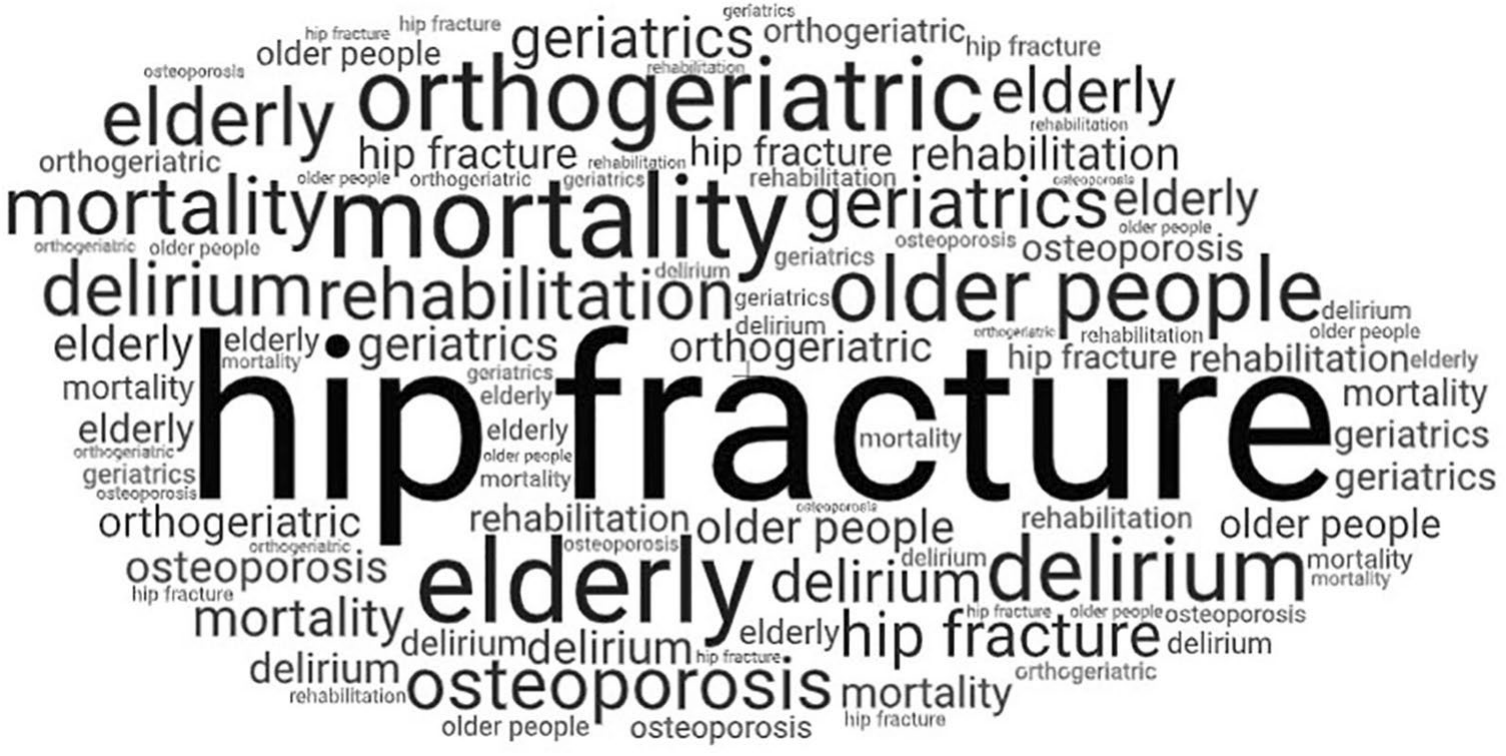
Word cloud showing the keywords used more than once. The keywords mostly used were “hip fracture”, “mortality” and “elderly” followed by “orthogeriatric” and “delirium”

The analysis of keywords showed that in total 93 words were used to describe the studies within the most cited 50 articles. The most frequently used keyword was “hip fracture” (used in 19% of the 50 most cited articles), followed by “mortality” (5%), “elderly” (5%), “orthogeriatric” (4%), delirium (3%), “older people” (3%), “osteoporosis” (2%), “geriatrics” (2%) and “rehabilitation” (2%). All keywords used more than once are depicted in Fig. 6.

**Fig. 7:**
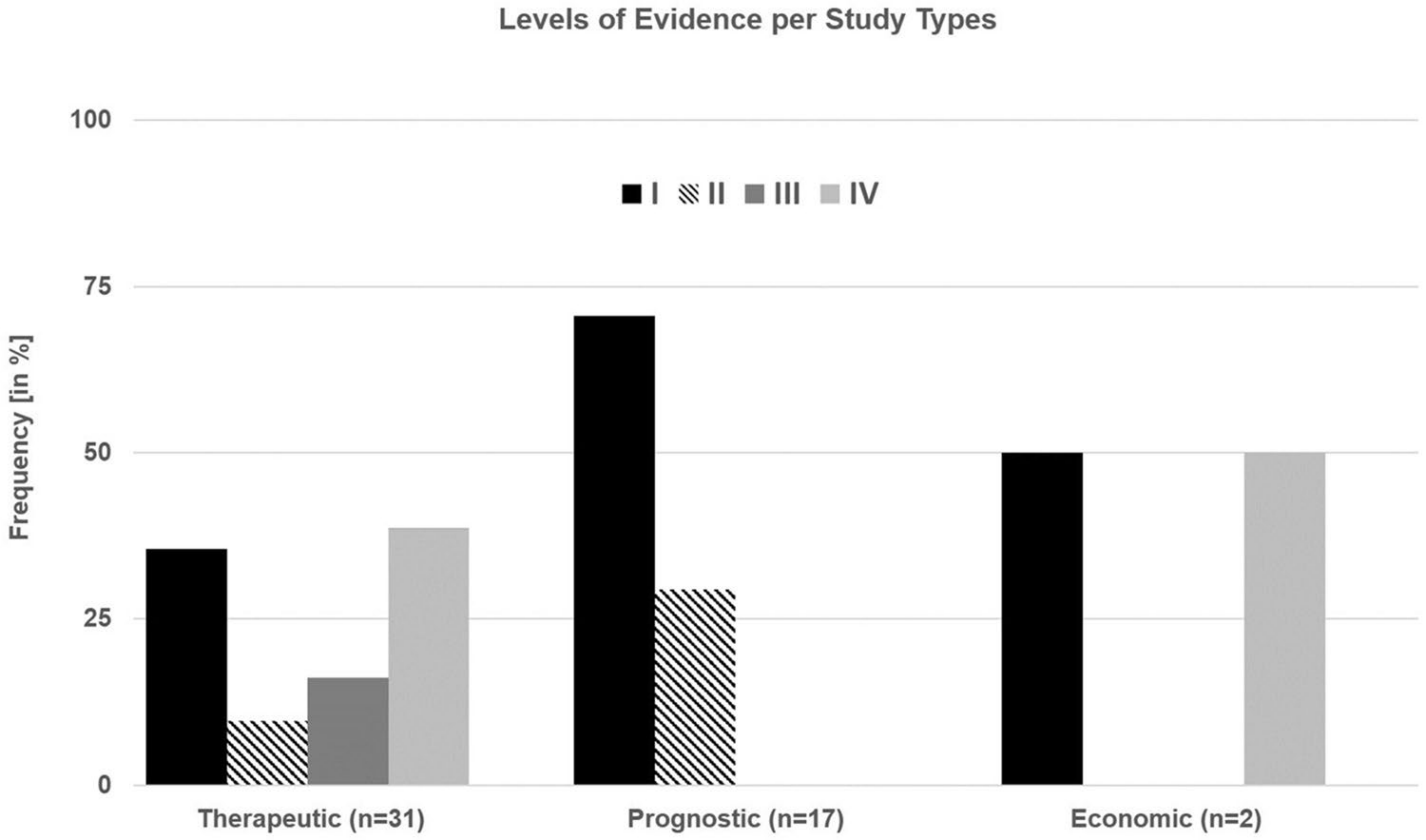
Graph showing the distribution of levels of evidence ranging from the highest (Level I) to lowest (Level V) per study type

The further analysis of study types showed the majority of articles reporting about therapeutic studies (62%), followed by prognostic studies (34%). Only two articles reported about economic and decision analyses; no articles with diagnostic studies were identified. The levels of evidence within these study categories are presented in Fig. 7. In detail, the distribution of levels of evidence were as follows: for (1) therapeutic studies *n* = 11 (35%) with level I, *n* = 3 (10%) with level II, *n* = 5 (16%) with level III, *n* = 12 (39%) with level IV, (2) prognostic studies *n* = 12 (71%) with level I, *n* = 5 (29%) with level II, (3) economic and decision analyses *n* = 1 (50%) each with either level I or level IV. Orthopaedic surgeons published none of the therapeutic articles with the highest level of evidence [16, 17, 25, 30, 39, 40, 42, 47, 51, 56, 57].

## Discussion

The population is ageing and health care professionals have to face new challenges of the orthogeriatric patient. Orthogeriatric care has been the new key topic within the last decades and continues at present in clinical research. Accordingly, the presented bibliometric review was performed to report about the “Status Quo” about existing literature on orthogeriatric care.

This bibliometric study demonstrates that research on orthogeriatric care is a high impact field in current clinical research with 50 top-cited research articles, with an average citation number of 93 citations per article. The differentiated analysis of authorship, origin and main subject of these articles reveals interesting insights. A small minority among the top-cited articles only are randomized controlled studies. Historic control group designs and prospective cohort studies for identification of prognostic factors find key interest. The finding of the increase of citations over time represents a logical consequence to the demographic change. The data provided by the United Nations (Population Division, Department of Economic and Social Affairs; World Population Prospects 2019) showed an increase in the population within 40 years (from 1980 to 2020; estimation, reference date as of 1 July) worldwide by factor 1.7, with an increase in the older population (minimum 65 years of age) by factor 2.8.

With regard to authorship, geriatricians were more often involved as first and last authors as compared to surgeons in highly cited articles. The types of journals also reflect this finding, as most articles were published in geriatric journals. In addition, there was a considerable number of key authors from disciplines other than surgery, such as epidemiology, endocrinology, or neuroscience. In fact, orthopaedic surgeons were first author in less than one-quarter of the articles. In addition, collaboration of orthopaedic surgeons with geriatricians was an infrequent finding. As a result, research groups without surgeons involved, rather than well-balanced interdisciplinary research groups performed and published the majority of studies. That indicates the potential to enhance an interdisciplinary approach to research. Orthopaedic surgeons should raise more frequently study questions related to perioperative management of older adults. The observation of the predominance of geriatrics in current research is likely related to the topics that were addressed in this research. Many studies explored geriatric co-management or geriatric aspects related to postoperative care, such as delirium, nutrition, rehabilitation. This demonstrates, that other topics, such as basic research, or clinical research on operative or anesthesiology procedures in geriatric orthopaedic patients might gain further interest in the future.

The analysis of keywords revealed that “hip fracture” was by far the predominant term used in the highly cited articles. Apparently, current research interest is mainly focused on orthogeriatric care of the geriatric patient with hip fracture. This focus on hip fracture care does not reflect the breadth of the field. Although hip fracture is one of the top diagnoses of geriatric orthopaedic patients, the majority of patients have other main diagnoses including fractures at other locations or elective surgery [58]. This indicates potential for further research areas; studies should neither be limited to the hip joint nor to fractures only. Alternatively other regions (e.g. spinal column, pelvis) or other pathologies (e.g. degenerative osteoarthritis) might be in the front in future.

Interestingly, the vast majority of the key cited articles have been conducted in Europe, and only a minority in other geographical regions. This finding was not expected. There are many active academic centres both in orthopaedic surgery as well as in geriatric medicine in North America, but less than 10% of highly cited articles were from this region. In addition, there is a remarkable level of heterogeneity of the origin of publications within Europe with high numbers of publications in selected European countries, and a lack of publications in other, mostly eastern European countries. Limited financial support might account for that finding, as the Gross Domestic Product per Capita was statistically significant lower in these countries compared to European countries being origin of identified articles. The five European countries with the highest number of publications (UK, Sweden, Spain, Norway, and France) were the origin of more than half of the 50 most cited articles. Asia accounts for the highest amount of the global older population, but the number of publications was considerably lower compared to Europe. A reason might be that in Europe the awareness for an orthogeriatric collaboration is higher than in other continents as in Europe the amount of older adults in relation to its entire population is highest compared to all other continents. Another issue might be that different availability of geriatric services within countries results in different scientific output in this field. In addition, the implementation of orthogeriatric care is a rather recent evolution with some countries just starting research in this field. In addition, a “pay-for-performance program” with a best practice tariff might be supportive indicating the role of the government in supporting orthogeriatric care and by that data acquisition suitable for orthogeriatric research [59].

## Limitations and strengths

A limitation might be that publications not indexed within Web of Science Core Collection are not included or the search strategy or the language might have limited the number of retrieved articles. The inclusion of only 50 articles might have excluded eminent articles. The citation count might be a measure of delay such as the study per se; accordingly, results might change once the search has been redone in the future. A further limitation might be that in the analysis of research original articles only, but review articles were not included. In addition, basic research or studies on the development of specific surgical procedures potentially contributing to the care of geriatric orthopaedic patients may have been missed, as those might be not being identified as “orthogeriatric literature”. A strenght of the study is the accessibility of the data without advanced statistical methods to provide understanding of current research topics of eminent literature in a relevant global challenge for health care professionals.

## Conclusion

Key countries (UK, Sweden, and Spain) and key topic (hip fracture) are key drivers in the orthogeriatric research. This bibliometric review acknowledges recent research but raises awareness that timely continuation and optimization for research in orthogeriatrics is necessary. Surgeons have a potential to contribute as regions or health care systems in which the topic has not been addressed so far. Other topics such as intraoperative procedures, fractures other than hip fractures or elective surgery are related topics with the potential for widening the field to research.
